# Nanovesicle‐Based Delivery of Magnesium Chlorophyllin for Photodynamic Inactivation in Agriculture

**DOI:** 10.1002/smsc.202500655

**Published:** 2026-05-08

**Authors:** Lisha Zhao, Wenzi Ckurshumova, Ava Ettehadolhagh, Jun Liu, Michael Fefer, Todd Hoare

**Affiliations:** ^1^ Department of Chemical Engineering McMaster University Hamilton Ontario Canada; ^2^ Suncor Energy Inc. Calgary Alberta Canada; ^3^ Centennial College Scarborough Ontario Canada; ^4^ Whitby Ag Consulting Whitby Ontario Canada

**Keywords:** agriculture, nanoparticles, nanovesicles, photodynamic inactivation, photosensitizer

## Abstract

Photodynamic inactivation offers a broad‐spectrum anti‐pathogen strategy for agriculture but requires effective delivery of the photodynamic activators to enable efficacy. Herein, we demonstrate that nanovesicles based on sodium dodecylbenzenesulfonate (SDBS) and cetyltrimethylammonium bromide (CTAB), prepared with modifiers to either enhance or reduce the stability of the bilayer membrane, can encapsulate and improve the functionality of magnesium chlorophyllin (Mg‐chl). SDBS/CTAB nanovesicles with sizes as small as ∼90 nm can be fabricated with encapsulation efficiencies of >60% for Mg‐chl. The incorporation of unsaturated modifiers into nanovesicle membranes enables triggered Mg‐chl release upon re‐wetting, whereas the introduction of hydrophobic moieties substantially slows Mg‐chl release via a more diffusion‐governed mechanism. Nanovesicles enabled substantially higher light‐activated killing of the plant pathogen *P. syringae* in simulated field conditions, with rapid binding and/or bacterial uptake observed within five mins of nanovesicle exposure. In addition, nanovesicles facilitated improved Mg‐chl penetration into plant roots. This combination of enhanced anti‐bacterial activity and tunable photosensitizer uptake offers promise to deliver photosensitizing agents or other functional hydrophilic bioactives for improved crop protection.

## Graphical abstract

Nanovesicles based on sodium dodecylbenzenesulfonate (SDBS) and cetyltrimethylammonium bromide (CTAB), prepared with modifiers to either enhance or reduce the stability of the bilayer membrane, can effectively encapsulate the photodynamic agent magnesium chlorophyllin (Mg‐chl) and enable both improved light‐activated bacterial eradication and enhanced root penetration for improved crop protection.

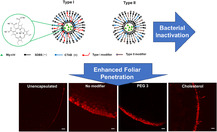

**Abbreviations**
aPDI: Antimicrobial photodynamic inactivation
CTAB: cetyltrimethylammonium bromide
CFU: colony forming units
CLSM: confocal laser scanning microscope
EE: encapsulation efficiency
Mg‐chl: magnesium chlorophyllin
PALS: phase analysis light scattering
PEG: polyethylene glycol
ROS: reactive oxygen species
SDBS: sodium dodecylbenzenesulfonate
TEM: transmission electron microscopy

## 1 Introduction

Antimicrobial photodynamic inactivation (aPDI) utilizes photosensitizing molecules that react with oxygen upon exposure to light to generate cytotoxic reactive oxygen species (ROS) that can oxidize a large range of biomolecules in cells, leading to severe damage of various structural components (e.g., membranes, organelles, and cytoskeleton) and thus near‐instantaneous microbial death [1, 2]. Because of its broad spectrum of action against fungi, bacteria, and viruses, including drug‐resistant strains, aPDI has attracted considerable interest in the medical field [3]. More recently, aPDI has attracted increasing attention in agricultural applications due to many of the same reasons; relatively few classes of currently available pesticides are effective against a broad set of pathogens [4], the treatment of resistant strains is becoming more challenging [5], and the broad range of damage imparted to bacteria by aPDI limits the chances that plants microbes can develop tolerance or resistance [6]. However, the fast photodegradation, limited solubility, and generally low bioavailability of most available photosensitizing molecules pose significant challenges in the application of aPDI in agriculture. More specifically, since the proximity of generated ROS to the pathogen is essential for function given that the short lifetime of ROS results in limited diffusion distances, both photodegradation and low solubility significantly decrease the probability that a photosensitizer can be brought close enough to the target pathogen to enable effective killing [7]. In addition, Gram‐negative bacteria are generally less susceptible to aPDI given that their impermeable outer membrane limits the penetration of anionic and neutrally‐charged molecules, typically requiring the co‐administration of membrane‐destabilizing agents such as metal chelators [8, 9], peptides [10] or cationic molecules [11, 12] to enable effective killing [13].

The challenges around photosensitizer use can be addressed in part by judicious photosensitizer selection. Among the most promising photosensitizers for agriculture in this context are cyclic tetrapyrroles derived from naturally occurring chlorophyll, as they can absorb a substantial portion of photons in the photosynthetically active range of the solar irradiance [14]. Sodium magnesium chlorophyllin (Mg‐chl, E‐140) is of particular interest given that it is a semi‐synthetic water‐soluble chlorophyll derivative that effectively generates singlet oxygen (^1^O_2_) under sunlight‐based activation [15, 16], enabling high killing efficacy against plant pathogenic bacteria and fungi in vitro. However, the practical application of Mg‐chl in the field is hindered by multiple factors: (a) Mg‐chl shows relatively low light and chemical stability, with activity losses of >90% observed in aqueous solutions after 6 h of sunlight exposure [17]. (b) Its water solubility is limited due to the inherent amphiphilicity of porphyrin molecules, significantly hindering its uptake and bioavailability in plant applications and limiting the dosing options available for effective utilization [18, 19]; and (c) the singlet oxygen from free Mg‐chl bioactive is generated rapidly upon activation, making it challenging to impart longer‐term antibacterial effects that are more compatible with a typical farmer's spraying schedule [20]. As such, the development of improved strategies for solubilizing, stabilizing, and delivering Mg‐chl is critical for its practical use in an agricultural context.

Engineered nanoparticles have emerged as an effective strategy to tackle multiple challenges around the effective utilization of both traditional and emerging bioactives in agriculture [21]. Nanoparticles can protect bioactives from chemical degradation under adverse environmental conditions [22], reduce the phytotoxicity of the bioactive [23], and improve the water dispersity of lipophilic or amphiphilic organic agrochemicals for enhanced uptake, bioavailability, and site‐targeted controlled delivery [23, 24]. A variety of different types of nanomaterials, including metallic nanoparticles (e.g., gold or silver) [26], oxidized metallic nanoparticles (e.g., MgO, CaO, ZnO, and TiO_2_) [27, 28, 29], carbon nanotubes [30, 31], poly(lactic‐co‐glycolic) acid nanoparticles [32], chitosan nanogels [33, 34], liposomes [35], and surfactant‐based based niosomes [36, 37], that have been reported to deliver various agrochemicals to control different plant diseases and/or improve plant growth. Nanovesicles, nanocarriers composed of amphiphiles such as phospholipids or surfactants self‐assembled to form a hydrophobic bilayer domain surrounding an aqueous core, have also been reported to deliver a variety of drugs, nutrients and bioactive compounds [38, 39]. Relative to other carriers, nanovesicles offer particular potential to co‐load bioactives that are both hydrophilic (in the aqueous core) and hydrophobic (in the bilayer) [40], a particular advantage for delivering a low‐solubility bioactive like Mg‐chl. Furthermore, the similar structure of nanovesicles to cell membranes can enhance effective nanoparticle uptake into cells, beneficial for increasing the bioavailability of the ROS production inside plants [41, 42]. Controlled disassembly under specific environmental conditions (e.g. light, heat or pH) depending on the components chosen for nanovesicle fabrication has also been reported [43], offering potential for targeted delivery in which the ROS‐generating species is released at an infected site.

**FIGURE 1:**
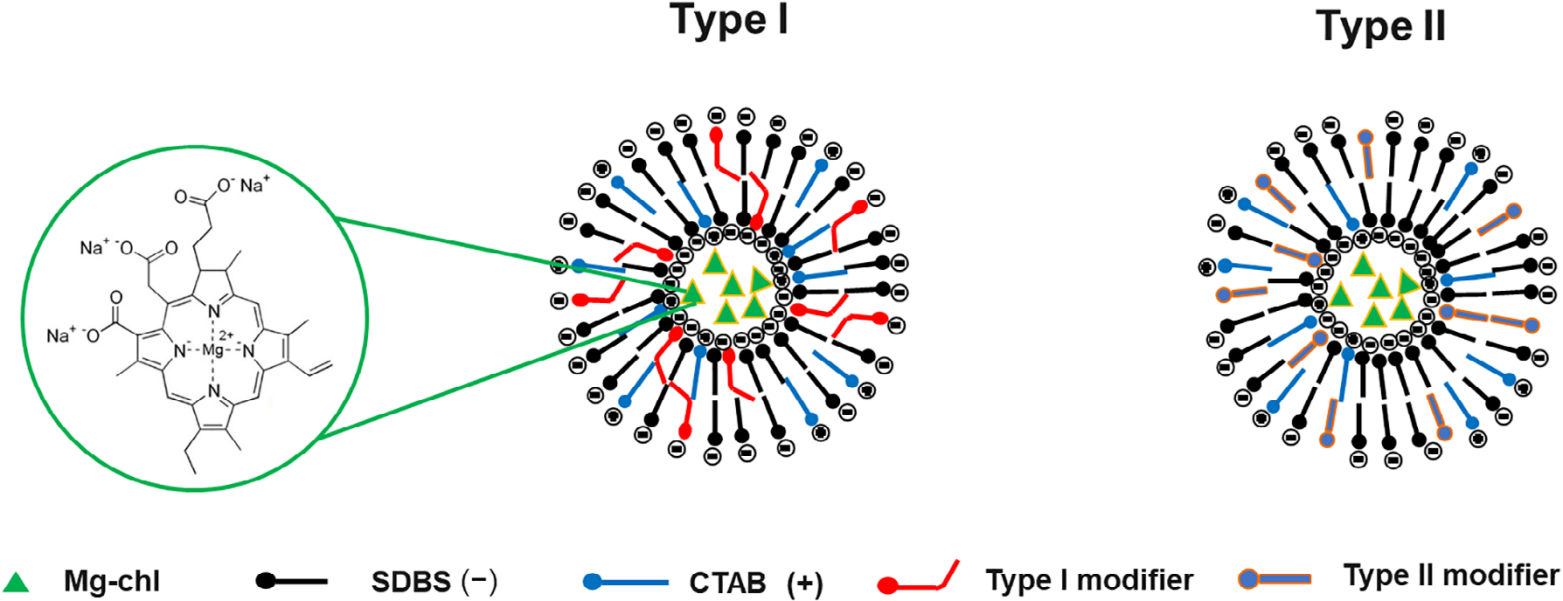
Schematic illustration of surfactant hybrid nanovesicles with two types of modification. Type I: the modifier is incorporated into nanovesicles to weaken membrane bilayer packing and thus enhance bioactive release; Type II: the modifier is incorporated into nanovesicles to enhance surfactant tail packing and thus reduce bioactive release.

We have previously reported the fabrication of surfactant hybrid nanovesicles based on anionic sodium dodecylbenzenesulfonate (SDBS) and cationic hexadecyltrimethylammonium bromide (CTAB), two commercial, highly water‐stable, and inexpensive surfactants, to deliver copper chlorophyllin to plants, showing improved photostability, sustained release, and significantly improved penetration of the bioactive into the plant as a result of nanovesicle encapsulation [44]. Herein, we adapt this strategy to develop modified hybrid nanovesicles for the effective encapsulation and delivery of Mg‐chl for photodynamic inactivation in agricultural applications (Figure 1). By tuning the stability of the nanovesicle membrane such that it either weakens (Type I) or strengthens (Type II) the stability of the bilayer membrane, we demonstrate the functionality of nanovesicles for plant uptake and the eradication of the Gram‐negative plant pathogen *Pseudomonas syringae* pv *tabacci* in simulated field conditions. We anticipate that SDBS‐CTAB hybrid nanovesicles can function as effective nanocarriers to both deliver photodynamically‐active Mg‐chl to plants over sustained periods and enhance penetration of the bioactive into plants, collectively enhancing the antimicrobial efficacy of Mg‐chl. More specifically, we demonstrate that surfactant‐based nanovesicles can encapsulate a photosensitizer without impeding the photodynamic properties of the active while also increasing the tissue penetration of the photosensitizer, providing multi‐functional benefits for enhancing the capacity of the photosensitizer for effective pathogen control.

## 2 Materials and Methods

### 2.1 Materials

Sodium magnesium chlorophyllin (Mg‐chl) was purchased from Organic Herb Inc. (Changsha, China). Sodium dodecylbenzenesulfonate (SDBS), cetyltrimethylammonium bromide (CTAB), cholesterol, Brij 10, and Tween 20 were obtained from Sigma–Aldrich (Oakville, ON, Canada). PEGylated phosphate esters (polyethylene glycol (PEG)‐3, 5 and 10) were provided by Croda Canada Ltd. (Vaughan, ON, Canada). Water purified using a MilliQ ultrapure water purification system (EMD Millipore, Billerica, MA, USA) was used for all experiments.

### 2.2 Preparation of Surfactant Hybrid Nanovesicles

The method for fabricating surfactant hybrid nanovesicles was adapted from our previous work [44]. SDBS and CTAB solutions (10 mM each) were separately prepared in 10 mM phosphate buffer adjusted to pH 7.5. Mg‐chl (0.1%, w/w) was fully solubilized in the SDBS solution under 300 rpm magnetic stirring for 15 min, after which the CTAB solution was added at an optimized mix molar ratio of 4:1 previously identified to avoid phytotoxicity with plants [44]. To fabricate modified nanovesicles, a modifier ingredient (PEGylated phosphate esters, cholesterol, Tween 20, or Brij C10) was subsequently mixed with the surfactant mixture at 300 rpm until all precursor surfactants were visually fully dispersed. The surfactant mixture was then homogenized at 9000 rpm (T25 digital ULTRA‐TURRAX, IKA Works Inc., Wilmington, NC, USA) for 7 min, after which it was immediately sonicated using a high‐intensity probe sonicator (Qsonica Q700, Newtown, CT, US) at a power of 700 W and a frequency of 20 kHz in an ice bath for 2 min (static interval: 2s) to create nanovesicles. Unloaded nanovesicles were fabricated using the same approach but excluding the Mg‐chl component. All nanovesicles were stored under nitrogen in glass vials in dark at 4°C, and tested within 1 week of fabrication.

### 2.3 Characterization of Nanovesicles

#### 2.3.1 Particle Size Distribution

The intensity‐weighted hydrodynamic diameter and polydispersity of the nanovesicles were determined using dynamic light scattering (Model 90 Plus, Brookhaven Instruments, Holtsville, NY, USA) operating at a laser wavelength of 640 nm and a scattering angle of 90°. The nanovesicle suspensions fabricated in section 2.2 were diluted 10× in 10 mM phosphate buffer (pH 7.5) and measured at 25°C. Five replicate measurements were conducted, with the error bars representing the standard deviation of those measurements.

#### 2.3.2 Zeta Potential

Nanovesicle zeta potential was measured using phase analysis light scattering (PALS) (Model 90 Plus, Brookhaven Instruments, Holtsville, NY, USA) by diluting the prepared nanovesicle suspensions 10× in 10 mM phosphate buffer and testing the samples at 25°C using a laser power of 60 mW and an electric field of 25 V/cm (*n* = 5 replicates per sample, with the error bars representing the standard deviation).

#### 2.3.3 Particle Morphology

The morphology of bioactive‐loaded nanovesicles was measured via transmission electron microscopy (TEM). The nanovesicle suspension was diluted 10‐fold in 10 mM phosphate buffer, after which one drop of the suspension was deposited onto a Formvar‐coated 200 mesh Cu/Pd grid and left to adhere for 10 min. Excess nanovesicle dispersion was carefully blotted dry from the edge and negatively stained using 50 μL of 1% (w/w) aqueous uranyl acetate for 20 s, after which excess stain was also removed by gentle blotting. Imaging was conducted using a JEOL JEM 1200EX TEMSCAN transmission electron microscope (JEOL, Peabody, MA, USA) operating at an accelerating voltage of 80 kV.

#### 2.3.4 Encapsulation Efficiency

The encapsulation efficiency (EE) of Mg‐chl into the nanovesicles was determined by the quantification of free and total bioactive content (µg) following encapsulation. To measure free (unencapsulated) bioactive, 1 mL of the surfactant nanovesicle dispersion was ultracentrifuged (Sorvall WX90+ ultracentrifuge, Thermo Fisher Scientific, Waltham, MA, USA) at 360,000 × *g* for 40 min at 4°C to separate the unencapsulated from the encapsulated bioactive. The supernatant (unencapsulated bioactive) was removed, diluted 20‐fold in a 10 mM phosphate buffer, and analyzed via UV spectrophotometry using a microplate reader (Infinite M1000 PRO, Tecan Group Ltd., Männedorf, Switzerland), tracking specifically the most prominent peak from the Mg‐chl absorbance spectrum at 400 nm [45]. To verify the peak wavelength, a full spectrum UV–vis scan of Mg‐chl was performed (Figure S1) that showed two absorption peaks at 400 and 654 nm; given the higher intensity of the 400 nm peak (enabling higher sensitivity in the concentration measurements), the 400 nm peak was selected as the measured absorbance wavelength. A calibration curve of absorbance versus free bioactive concentration was used to determine the concentration of free bioactive (µg/mL). The EE was measured immediately after the nanovesicles were formed on freshly prepared samples daily to exclude any potential complicating effects associated with the degradation of Mg‐chl in the aqueous phase. The experiment was performed in triplicate, with the error bars corresponding to the standard deviation of the replicate samples. The encapsulation efficiency (EE) in nanovesicles was determined according to Equation (1)

(1) $$\mathrm{EE}\;(\%)\;=\frac{\mathrm{Total}\;\mathrm{bioactive}\;\mathrm{content}\;(\mathrm{\mu g})‐\mathrm{Free}\;\mathrm{bioactive}\;\mathrm{content}\;(\mathrm{\mu g})}{\mathrm{Total}\;\mathrm{bioactive}\;\mathrm{content}\;(\mathrm{\mu g})}\;\times 100\%$$

#### 2.3.5 In Vitro Bioactive Release

In vitro Mg‐chl release kinetics were measured across multiple rehydration/release cycles. Encapsulation and measurement of EE% was performed as described in Section 2.3.4, after which the pellet was resuspended in 150 mL 10 mM phosphate buffer and incubated at ambient temperature for 2 h under gentle shaking to fully disperse the nanovesicle pellet. Following complete redispersion of the pellet, the suspension was separated into triplicate 5 mL aliquots and incubated without shaking at room temperature to mimic agricultural application. Because bioactive release started immediately right after phosphate buffer addition, bioactive release was recorded at the beginning of pellet resuspension step. At each pre‐determined time interval, the aliquot suspensions were ultracentrifuged at 360 000×g for 40 min at 4°C, a 4.7 mL sample of supernatant was removed, and the supernatant concentration of bioactive released Mg‐chl was analyzed via UV/vis spectrophotometry as described in Section 2.3.3. For additional time points, the nanovesicle pellet was either resuspended in the same supernatant (simulating continuous release in closed system, i.e., on a leaf without irrigation or rainfall) or suspended in fresh phosphate buffer (simulating re‐wetting of the formulation on the leaf upon irrigation or rainfall); at least three such release cycles were performed per release experiment to enable comparisons between samples. The experiment was performed in triplicate, with the error bars corresponding to the standard deviation of the replicate samples. The released bioactive concentrations were calculated according to Equation (2):

(2) $$\mathrm{Bioactive}\;\mathrm{release}\;(\%)\;=\frac{\mathrm{Release}\;\mathrm{bioactive}\;\mathrm{content}\;\left(\mathrm{\mu g}\right)}{\mathrm{Total}\;\mathrm{encapsulated}\;\mathrm{biactive}\;\mathrm{content}\;\left(\mathrm{\mu g}\right)}\;\times 100\%$$

#### 2.3.6 Bacterial Membrane Affinity Test

A 100 μL suspension of *Pseudomonas syringae* pv *tabacci* at a concentration of 10^8^ cells/mL was spread on Luria broth agar plates and cultured at 28°C for 48 h. 100 μL of Mg‐chl‐loaded nanovesicle suspension was placed on the bacteria for 5 min, after which the samples were washed with a PBS buffer to remove non‐associated nanoparticles. Bacteria were subsequently visualized using the Cy5 filter with an Olympus FV1200 confocal laser scanning microscope (CLSM) equipped with an oil immersion objective (60 ×, NA = 1.40), with all images recorded using a QICAM Fast 1394 digital camera (QImaging, Surrey, BC, Canada) and processed using Image‐Pro Plus 7.0 software (Media Cybernetics Inc., Rockville, MD, USA). Images of the bacterial cells after nanovesicle treatment were recorded using CLSM to assess the interaction between Mg‐chl and bacteria.

#### 2.3.7 DNA Release Study

The cellular DNA treated by nanovesicles was evaluated following the method reported by Marino et al. [46]. To assess the release of cellular DNA from Gram‐negative *Pseudomonas syringae* pv *tabacci* upon exposure to free and nanovesicle‐loaded Mg‐chl, 1 mL of a 10^8^ cells/mL *P. syringae* suspension was incubated in the presence or absence of free Mg‐chl or encapsulated nanovesicles (maintaining an overall concentration of 100 μM Mg‐chl each sample) for 30 min at 28ºC, after which the samples were exposed to light (2000 µmol·m^−2^ s^−1^ light intensity) for 1 h using a programable LED System (RX 30, Heliospectra, San Rafael, CA, USA). After light exposure, the bacteria were centrifuged for 2 min at 10 000 rpm, and the concentration of released DNA in a 2 μL supernatant was quantified by measuring the absorbance at a wavelength of 260 nm using a NanoQuant Plate (Infinite 200 PRO, Tecan, Switzerland) [46, 47]. Control samples were evaluated in a similar manner, except that the samples were stored under dark conditions for 1 h before the quantification of DNA. All measurements were performed using 15 replicates and normalized against the corresponding sample at starting time of 0 s.

#### 2.3.8 Antibacterial Efficacy Test

The antibacterial activity of Mg‐chl against *Pseudomonas syringae* pv *tabacci* was evaluated by mixing 20 µL of 10^8^ cells/mL *P. syringae* suspension supplemented with 5% soil load (fetal bovine serum) with 20 µL of free or encapsulated Mg‐chl nanovesicles (0.1% (w/w) Mg‐chl loading) and pipetting the mixture on a sterile polystyrene plate using a similar method to that reported by [48]. The droplet was exposed to a LED light system (RX 30, Heliospectra San Rafael, CA, USA) at ambient temperature for 1 h until completely dry. The dry drop was then resuspended in 100 µL of 100 mM sodium phosphate buffer, serially diluted, plated in 100 μL aliquots on LB agar plates, and incubated at 28°C for 48 h. After the incubation, colonies were counted and the viable bacteria concentration was reported as the number of colony forming units per mL of sample (CFU/mL). Antibacterial efficacy measurements were performed in triplicate, with the error bars corresponding to the standard deviation of the replicate samples.

#### 2.3.9 In Planta Uptake Test

The in planta uptake of lead nanovesicles was evaluated by confocal laser scanning microscopy. To avoid chlorophyll autofluorescence in green leaves, we visualized uptake in intact soybean roots. Soybean seedlings were germinated in the dark on wet paper towels for 7 days. Roots were dipped in 10 mM nanovesicle formulations in sodium phosphate buffer for 1 h, washed thoroughly with Milli‐Q water, and hand sectioned. Sectioned roots were placed in the centre of one drop of Milli‐Q water on a glass slide and imaged with an Olympus FV1200 confocal microscope (Olympus Italia Srl, Milan, Italy), using an excitation wavelength of 488 and 680 nm. All images were recorded using a QICAM Fast 1394 digital camera (QImaging, Surrey, BC, Canada) and processed using Image‐Pro Plus 7.0 software (Media Cybernetics Inc., Rockville, MD, USA).

## 3 Results and Discussion

### 3.1 Nanovesicle Physicochemical Properties

**FIGURE 2:**
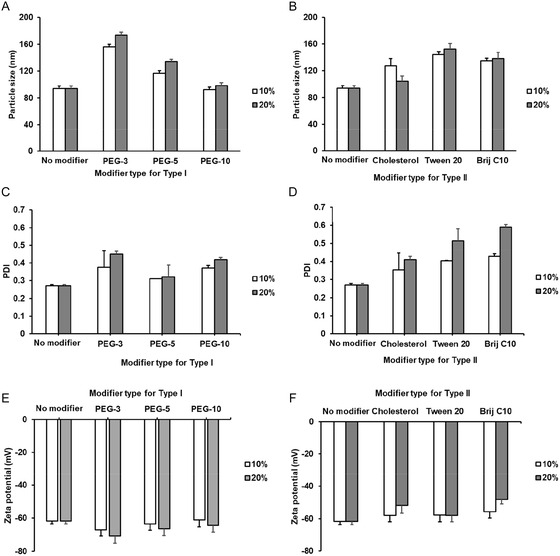
Physical characterization of modified SDBS/CTAB nanovesicles: (A–D) (A,B) Particle size and (C,D) polydispersity of SDBS‐CTAB nanovesicles prepared using (A,C) Type I modifiers or (B,D) Type II modifiers at modifier concentrations of; (E,F) Zeta potential of SDBS‐CTAB nanovesicles prepared using (E) Type I modifiers or (F) Type II modifiers at modifier concentrations of 10 mol% (unfilled series) and 20 mol% (filled series) relative to the total SDBS/CTAB concentration. All nanovesicles were loaded with 0.1% (w/w) Mg‐chl.

To facilitate scaled‐up processing of nanovesicles, only homogenization and sonication steps were used to prepare nanovesicles, avoiding membrane extrusion or microfluidic techniques often used to prepare liposomes and other nanovesicle systems [49]. Nanovesicles were prepared by mixing the base SDBS/CTAB surfactant combination (which facilitates both amphiphile self‐assembly as well as electrostatic interactions between the surfactants to enhance nanovesicle stability) with two different types of additives: (1) Type I additives (PEGylated phosphate esters with different PEG chain lengths and unsaturation in the hydrophobic tail) selected to disrupt the packing of SDBS/CTAB and increase membrane permeability; and (2) Type II additives (cholesterol, Tween 20, and Brij C10) selected to enhance the packing of SDBS/CTAB amphiphiles within the bilayer and reduce membrane permeability. Note that the nanovesicles were not purified to remove non‐assembled surfactant, consistent with the likely practical use case for nanovesicles in large‐scale agrochemical delivery applications in which secondary purification processes pose an economic barrier to translation. The particle size, size polydispersity, encapsulation efficiency, and zeta potential of 0.1% (w/w) Mg‐chl‐loaded nanovesicles prepared with each of the two modifier types are shown in Figure 2. Modifiers of both types generally increased the particle size and the polydispersity relative to modifier‐free SDBS‐CTAB nanovesicles, consistent with previous reports with other nanovesicles [50, 51]. However, the reasons for such size changes vary depending on the nature of the modifier; Type I modifiers disrupt the lipid packing and increase the fluidity of membrane bilayers, while the Type II modifiers increase the hydrophobic packing volume of the surfactants [52, 53]. However, the degree of particle size and polydispersity changes observed differed between the different modifiers. For Type I modifiers, as the length of the poly(oxyethylene) hydrophilic chain was increased, the particle size of nanovesicles was significantly reduced from 179 nm (PEG‐3) to 92 nm (PEG‐10), the latter matching the particle size of the modifier‐free SDBS‐CTAB nanovesicles. The longer PEG chain length of the Type 1 modifier provides improved steric stabilization of the nanovesicle in addition to the electrostatic stabilization provided by SDBS/CTAB, enabling the stabilization of more total surface area (i.e., smaller particles) that are less likely to aggregate/fuse with each other [54, 55, 56]. Note that CTAB can induce phytotoxicity in plant studies at higher concentrations [44]; diluting the CTAB content without compromising the size or stability of the resulting nanovesicle thus may have benefits in practical use cases of the nanovesicles to increase bioactive delivery without creating phytotoxicity challenges. Correspondingly, relatively modest increases in polydispersity were observed with the Type 1 modifiers, with most formulations showing polydispersities in the 0.3–0.4 range relative to the 0.27 polydispersity observed with modifier‐free nanovesicles; the PEG‐5 formulation showed particularly modest polydispersity increases. Interestingly, the zeta potential did not significantly change upon the addition of even up to 20 mol% Type 1 modifier despite the PEG‐based modifiers being electrostatically neutral and thus in principle serving to dilute the charge from the SDBS/CTAB surfactants. We hypothesize this result is correlated to changes in ion permeability within the micelles as the Type 1 modifiers reduce the rigidity of the membrane, increasing the electrophoretic softness to counteract any effect of the modifiers reducing the net surface charge density on the nanovesicle zeta potential measurements [57]. On the other hand, Type II‐modified nanovesicles all exhibited significant increases in particle size relative to the modifier‐free nanovesicles coupled with significantly broader particle size distributions (Figure 2d), with the Brij C10‐modified nanovesicles in particular showing high polydispersity (0.45–0.6); however, the DLS correlegrams indicated a good fit to the correlation function, suggesting these values can be accurately reflected by the DLS measurements (Figure S2). All Type II nanovesicles also maintained highly anionic surface charges with a similar zeta potentials at 10 mol% modifier content (∼**−**60 mV). However, the absolute value of the zeta potential decreased for the cholesterol and Brij C10‐modified nanovesicles when the modifier content was increased to 20 mol%, potentially due to (1) the role of the neutral Type II additives in increasing the membrane stiffness (reducing electrophoretic softness) while simultaneously reducing the net surface charge and/or (2) the presence of a larger particle size fraction due to the observed broadening in the polydispersity of the Type II‐modified nanovesicles. However, given that minimal size/polydispersity/charge differences overall were observed between 10 and 20 mol% modifier nanovesicles in each sample, the 20 mol% modified nanovesicles were used for subsequent analysis to more unambiguously identify potential modifier effects on nanovesicle performance.

### 3.2 Nanovesicle Morphology

**FIGURE 3:**
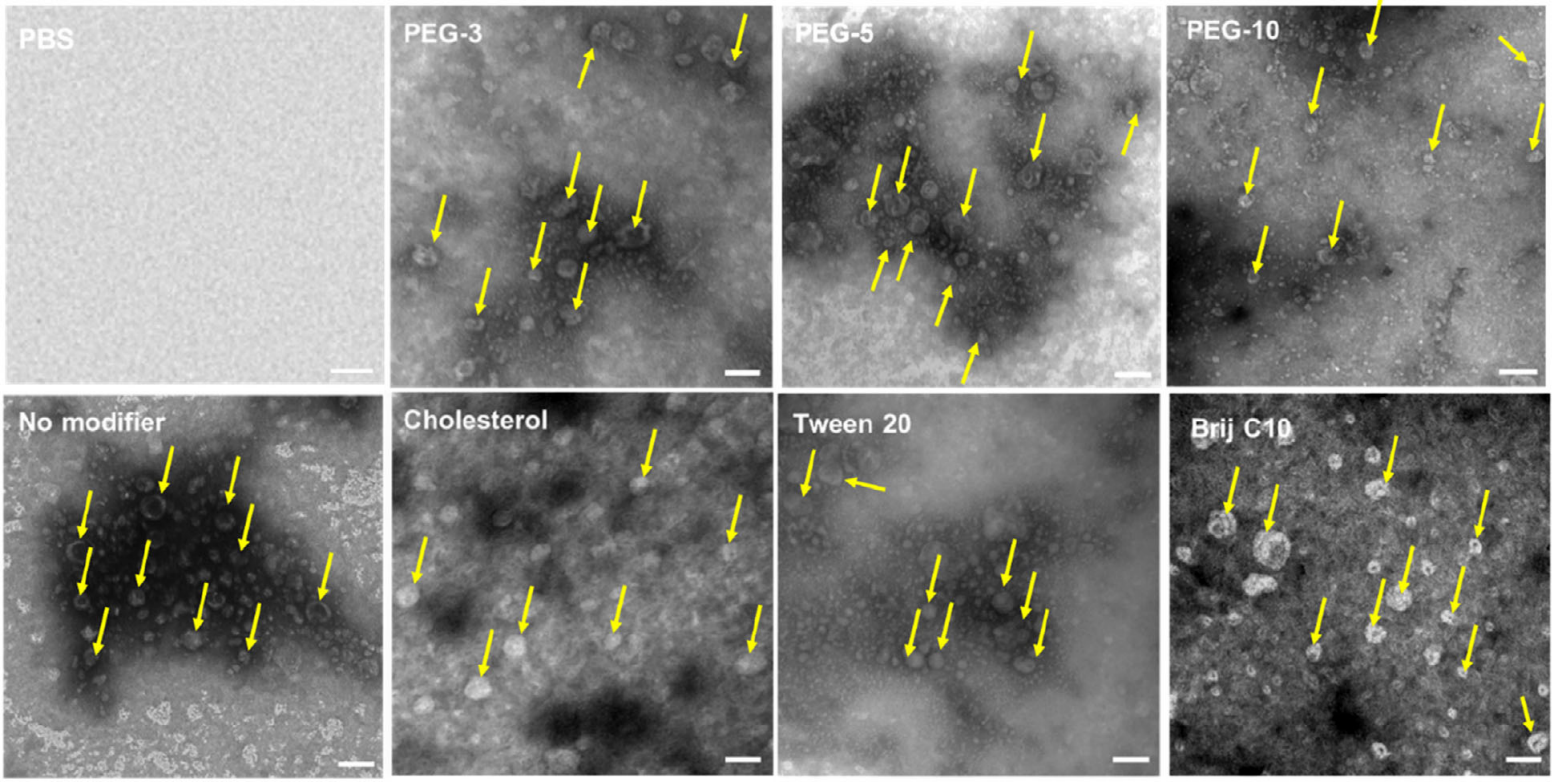
TEM images of modifier‐free and modified SDBS‐CTAB nanovesicles prepared using 20 mol% of Type I (top row) and Type II (bottom row) modifiers. All nanovesicles were loaded with 0.1% (w/w) Mg‐chl. Scale bars = 100 nm in each image. Yellow arrows point to representative identified nanovesicles in each image for clarity.

The morphologies of SDBS‐CTAB nanovesicles prepared with or without modifiers loaded with 0.1% (w/w) Mg‐chl were visualized via transmission electron microscopy (TEM), with representative images for each formulation shown in Figure 3. All modifiers produced nanovesicles of a generally spherical shape with relatively heterogeneous populations, with the visual particle sizes and size distributions directly correlating with the relative DLS‐measured particle sizes shown in Figure 2 (albeit with the TEM‐measured particle sizes being consistently smaller due to the dehydration of the nanovesicle required for TEM sample preparation and analysis) (Table S1). More specifically, modifier‐free and Type I‐modified nanovesicles showed significantly better uniformity than Type II Tween 20 and Brij C10‐modified nanovesicles. The darker staining inside the majority of the nanovesicles indicates efficient diffusion of the water‐soluble TEM contrast dye into the nanovesicles, consistent with the proposed hollow structure of the nanovesicles desirable to enable the efficient loading of water‐soluble bioactives such as Mg‐chl [44]. However, the lack of visible dye penetration inside the Type II cholesterol and Tween 20‐modified nanovesicles suggests enhanced packing and thus reduced bilayer permeability in these nanovesicles, consistent with the findings of [58] that Tween 20 and cholesterol enhanced the lipid order and membrane elasticity and thus improved the stability of bilayer membranes. Conversely, PEG‐10 and Brij C10‐modified nanovesicles appear collapsed and somewhat distorted in the TEM image, suggestive of lower membrane elasticities of these samples relative to those prepared with other modifiers. For PEG‐10, the reduced membrane stiffness is likely attributed to the modulated packing of the bilayer membrane and thus the destabilizing effect induced by the increased number and flexibility of PEG units [59, 60]. Analogously, Brij C10 has the same number (10) of PEG units as PEG‐10 and has been reported to disturb lipid membranes [61] and thus increase the chance of defect and pore formation on nanovesicles [62].

### 3.3 In Vitro Bioactive Loading and Release

**FIGURE 4:**
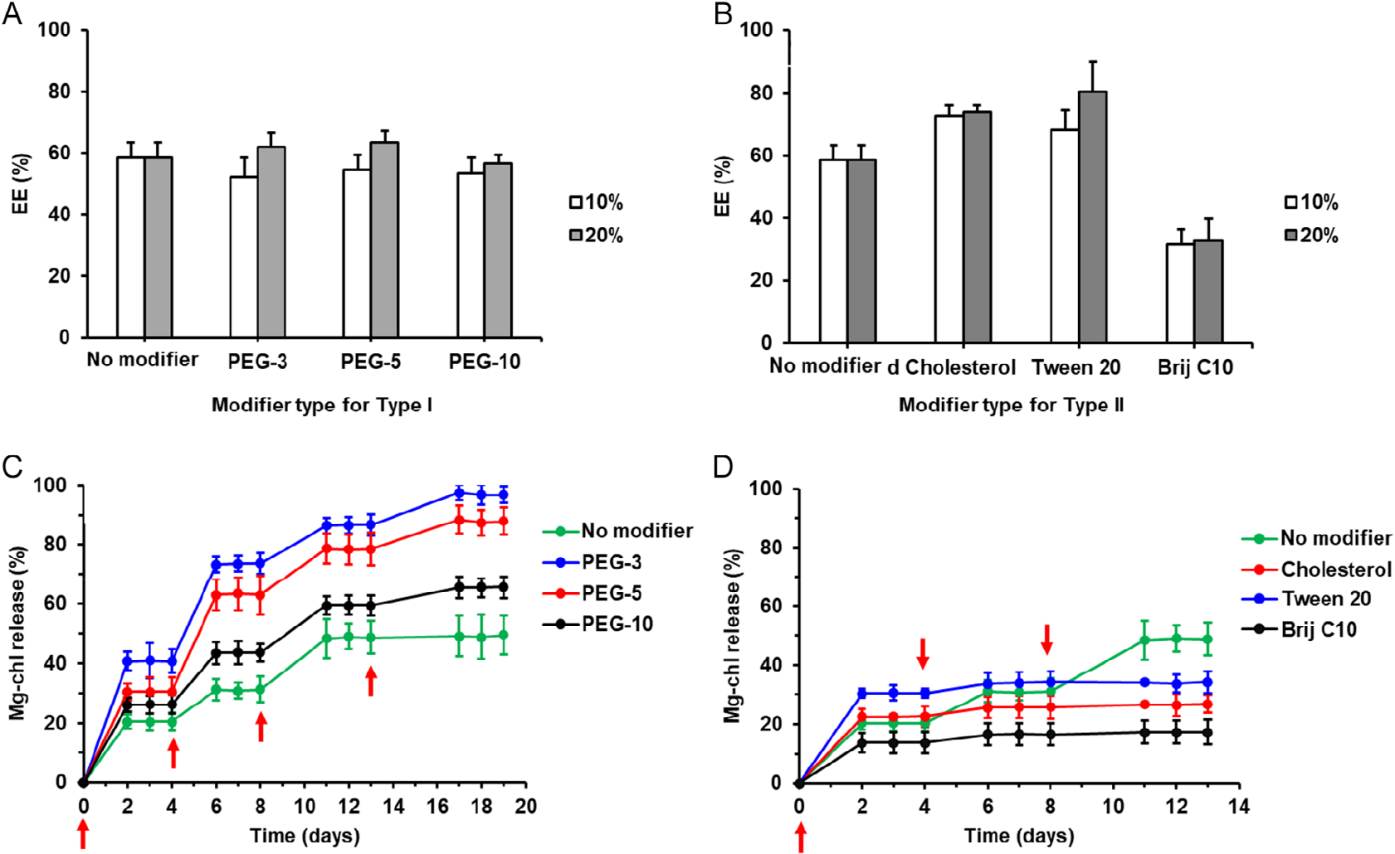
(A,B) Encapsulation efficiency and (C,D) subsequent release kinetics of Mg‐chl‐loaded SDBS‐CTAB nanovesicles prepared with or without Type I or Type II modification: (A,B) Encapsulation efficiency of Type I (A) and Type II (B) modified nanovesicles prepared at different modifier mole fractions; (C,D) In vitro release kinetics from 20 mol% modifier nanovesicles measured in 10 mM PBS solution (pH 7.5). The Mg‐chl concentration was controlled at 0.1% (w/w). The arrows represent time points at which the supernatant following centrifugal separation of the nanovesicles was completely replaced with fresh 10 mM PBS solution instead of the nanovesicles being redispersed in the supernatant from the previous step.

The encapsulation efficiencies of Mg‐chl in both modifier‐free and Type I and Type II‐modified nanovesicles are shown in Figure 4. Modifier‐free SDBS‐CTAB nanovesicles encapsulated ∼60% of the initially added 0.1 w/w% Mg‐chl, a relatively high encapsulation efficiency for a self‐ assembled nanovesicle‐based system [64, 65]. We speculate that the potential for the cationic CTAB moieties in the bilayer to bind to anionic Mg‐chl (which has ∼3 anionic ‐COO^‐^ groups per molecule at pH 7.5) contributes to this high encapsulation efficiency observed. Type I‐modified nanovesicles showed similar EE values (53%–63%) to the modifier‐free system, with the slight dilution of the overall CTAB concentration observed likely offset by the high hydrogen bonding capacity of PEG that can also support Mg‐chl uptake. Except for the Brij C10‐based nanovesicles noted to have particularly large sizes/polydispersities (Figure 2) and relatively poor membrane stabilities (Figure 3), the other two Type II modifiers demonstrated higher encapsulation efficiencies for Mg‐chl (74% ± 2% for cholesterol and 80% ± 9% for Tween 20) consistent with the role of the saturated hydrophobes in these modifiers ultimately strengthening the bilayer packing and thus facilitating enhanced bilayer stability and bioactive retention [66, 67]. These high observed loading efficiencies are beneficial both to increase the amount of bioactive delivered per unit mass of nanoparticle (maximizing dose while minimizing cost/additive administration) and facilitate improved photoprotection for photolabile Mg‐chl, enabling prolonged efficacy as well as improved residual bioactivity.

The in vitro release of pre‐loaded Mg‐chl from modified nanovesicles over multiple release cycles in pH 7.5 PBS buffer is shown in Figure 4C for Type I modified nanovesicles and Figure 4D for Type II modified nanovesicles. Release samples were collected under two conditions: (1) the release media were sampled but not replaced (times not denoted by an arrow) and (2) the release media were fully replaced following ultracentrifugation to isolate the nanovesicles (times denoted by an arrow). Compared to modifier‐free nanovesicles (49% cumulative release after three buffer exchange cycles), Type I modification resulted in significantly accelerated Mg‐chl release (66%–97% cumulative release after three buffer exchange cycles), with release increasing as the PEG chain length was decreased. Conversely, Type II modification resulted in significantly slower release (17%–34%) relative to the modifier‐free nanovesicles. This difference between the two modifier types is consistent with the structure of the modifiers; alkyl chain unsaturation in the Type I modifiers disrupts the packing of the SDBS/CTAB to reduce membrane rigidity, thus increasing membrane permeability, while the saturated hydrocarbon chains of the Type II modifiers improve the packing and ordering of SDBS and CTAB amphiphiles and thus reduce membrane permeability [68, 69]. This interpretation is also consistent with known strategies to modulate drug release from liposomes [70, 71, 72]. Furthermore, the shape of the kinetic profiles within a single cycle (i.e., nanovesicles are re‐dispersed in the previous supernatant) versus between cycles (i.e., nanovesicles are re‐dispersed in fresh supernatant) is notable, particularly for the Type I modifiers with leakier membranes. In such cases, release of Mg‐chl is essentially pulsatile, with the diffusive release observed within each single cycle saturating the release media followed by a plateau until fresh buffer is added and the partitioning equilibrium is reset. This result suggests the potential for rain/irrigation‐triggered "on–off" release of Mg‐chl over multiple re‐wetting cycles, a potentially beneficial property for agricultural delivery over prolonged time periods. Alternatively, for Type II modifiers, a slower and more sustained release profile is observed following the initial sampling period (which includes desorption of externally‐bound Mg‐chl into the receiving solution), suggesting a more diffusion‐controlled release profile. Of note, release from nanovesicles prepared with Type II modifiers reached a plateau after 3 cycles, with no significant increase in the bioactive release observed during the 4th cycle (13–19 days). As such, depending on the type of modifier used, release can be accelerated or decelerated versus the modifier‐free nanovesicles. Note that all these results assume the nanovesicles would remain intact following foliar application; it is however, likely that the waxy layer of the leaf will disassemble or reconform the nanovesicles upon drying of the spray droplet, which may alter the kinetics of the bioactive release.

### 3.4 Functionality of Nanoparticles

#### 3.4.1 Nanovesicular Interactions With Bacterial Cells

**FIGURE 5:**
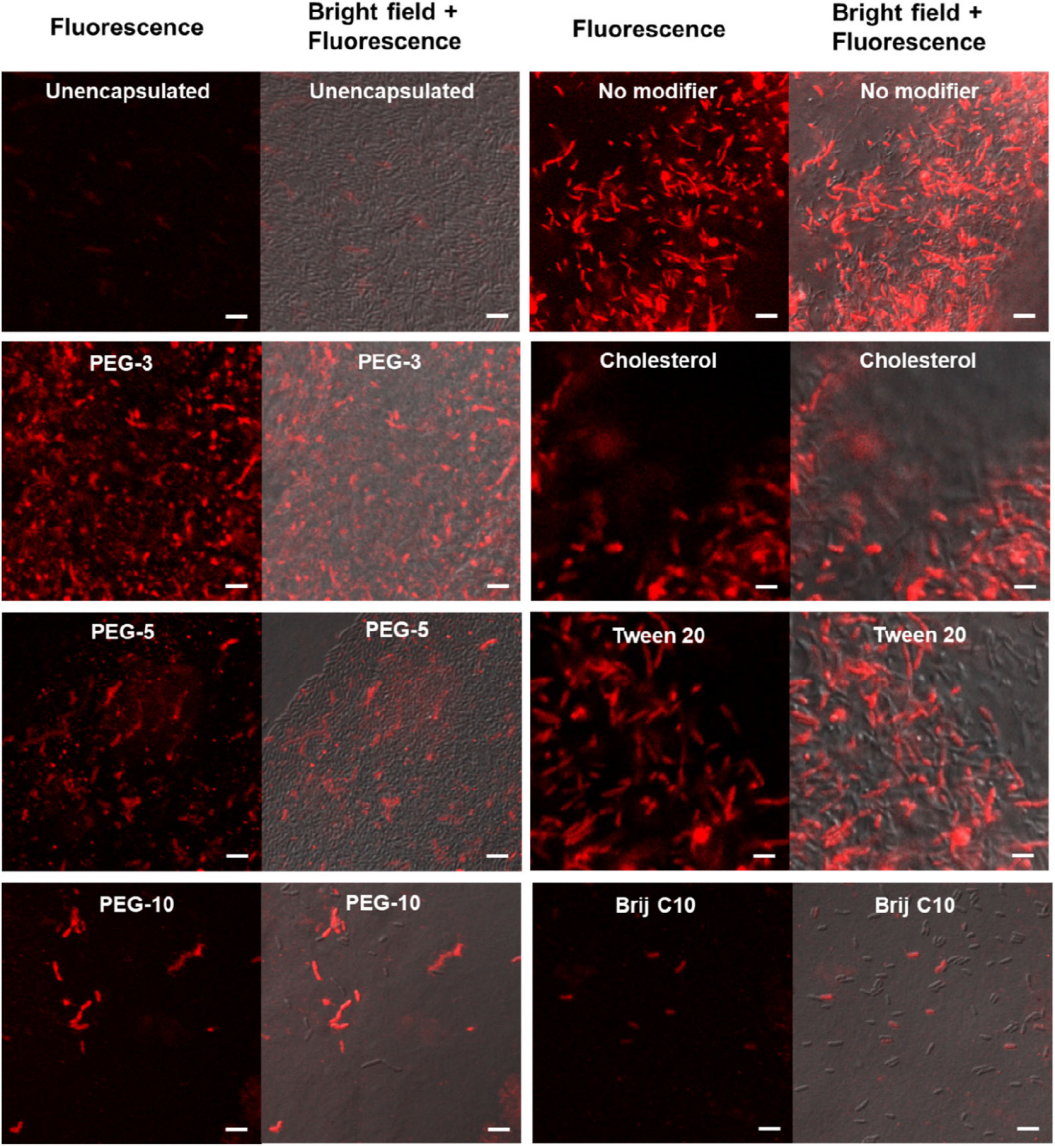
Confocal images of the interactions between Gram‐negative *Pseudomonas syringae* pv *tabacci* free with unencapsulated Mg‐chl (top left) relative to SDBS‐CTAB nanovesicles loaded with the same concentration of Mg‐chl (0.1% (w/w)) without added modifiers (top right) and with added Type I and Type II modifiers (20 mol% modifier concentration). The red fluorescence indicates the presence of Mg‐chl (488 nm excitation wavelength). Scale bars = 10 μm.

Efficacy of photodynamic inactivation can be influenced by the photosensitizer's proximity to the pathogen due to the limited range of ROS activity. To investigate how different nanovesicle formulations interact with bacteria, we visualized the association of nanovesicles prepared with different modifiers with the Gram‐negative plant pathogen *Pseudomonas syringae pv tabaci* using the natural fluorescence of Mg‐chl. Figure 5 shows confocal imaging of the localization of Mg‐chl with bacteria following short‐time exposures between the bacteria and the nanovesicle formulations. Free Mg‐chl showed minimal association with bacteria, consistent with its water solubility and high anionic charge that would be repelled by bacterial surfaces. In contrast, modifier‐free SDBS‐CTAB nanovesicles demonstrated strong and uniform fluorescence signals that overlapped strongly with optical images of the bacteria, suggesting that these nanovesicles interacted with the bacteria. Since Gram‐negative bacteria are coated with anionic lipopolysaccharides that carry a negative charge, although the nanovesicles also have a net anionic charge, the cationic CTAB component of the nanovesicles may undergo rearrangements to enhance binding and/or promote fusion or uptake of the nanovesicles into bacteria. Again, aside from the poorly‐defined Brij C10‐modified nanovesicles, all the Type I and Type II modified nanovesicles also showed enhanced affinity and interaction with the bacterial surfaces relative to the free Mg‐chl control. However, a clear trend in affinity was observed in the Type I modified group depending on the length of the PEG chain present in the modifier, with the incorporation of longer PEG chains significantly reducing the nanovesicle‐bacteria interactions. We speculate this result is a result of steric interactions between the PEGylated nanovesicles and the bacteria that may slow the kinetics of nanovesicle‐bacteria interactions relative to nanovesicles exclusively stabilized by charge (modifier‐free) [73], interactions that are enhanced when the PEG chain length is increased. The inclusion of Type II modifiers, cholesterol and Tween 80, in the nanovesicles also appeared to result in significantly larger fluorescent domains being present in the images, suggesting the possibility that the adhesion of one nanovesicle to the bacterial surfaces may make these more hydrophobic nanovesicles more prone to self‐aggregation on the bacterial surface. Such co‐localization of nanovesicles may be of benefit for concentrating the photosensitizer effect at a particular location on the bacterial membrane. However, in all cases, encapsulation of Mg‐chl into nanovesicles facilitated significantly enhanced interactions with bacteria relative to free Mg‐chl, suggesting potential benefits for bacterial photoinactivation.

#### 3.4.2 DNA Release

**FIGURE 6:**
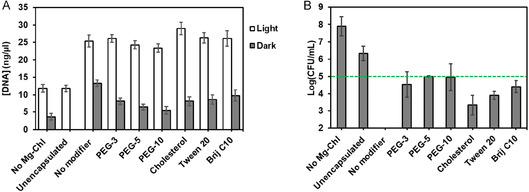
Photodynamic inactivation of *Pseudomonas syringae* pv. *tabaci* following treatment with unencapsulated Mg‐chl or Mg‐chl‐loaded SDBS‐CTAB nanovesicles with or without modification with Type 1 of Type 2 modifiers added at 20 mol% total surfactant concentration (0.1% (w/w) Mg‐chl loading in all tested samples aside from the negative control): (A) Cellular DNA release measured after 1 h incubation in the dark (gray bars) or under sunlight‐mimicking irradiation (white bars); (B) Antibacterial activity of free and encapsulated Mg‐chl on surface‐dried *P. syringae* as measured via colony counting. The green dashed line represents the effective disinfection threshold (3 log reduction of CFU count).

To assess the potential of Mg‐chl‐loaded nanovesicles to disrupt Gram‐negative bacterial membranes based on their demonstrated ability to associate with bacterial surfaces, DNA release from bacterial cells was measured with or without incubation with nanovesicles, both in the dark (no photoactivation) and upon light irradiation (with photoactivation), with the results summarized in Figure 6A. Free Mg‐chl did not induce significantly higher DNA release and thus membrane disruption relative to the bacteria‐only control, consistent with previous reports highlighting the resilience of Gram‐negative bacteria to aPDI with anionic porphyrins [74]; similarly, all unloaded nanovesicles induced only modest DNA release when stored in the dark, suggesting that the nanovesicles themselves have limited toxicity to bacteria in the absence of the photosensitizer. However, it is notable that the modifier‐free nanovesicle induced significantly more DNA release than the modified nanovesicles, suggesting that the role of the modifiers in diluting the CTAB concentration in the nanovesicle (which itself has noted antibacterial activity) may reduce the inherent toxicity of the nanovesicles themselves [75, 76]. Upon light activation, all nanovesicle formulations showed 2–3× higher DNA release relative to the corresponding sample in the dark and relative to free Mg‐chl. We hypothesize this finding can be attributed to the improved affinity and interaction of Mg‐chl‐loaded nanovesicles with bacterial cells (Figure 5), allowing for ROS production in closer proximity to the cell membrane under light exposure and thus improved capacity to oxidize membrane and cytoplasm components [77]. On this basis, the nanovesicles can effectively replace the membrane‐destabilizing agents typically required to enable effective aPDI of Gram‐negative bacteria [78]. Of interest, the degree of DNA release was not clearly correlated with the observed release kinetics of Mg‐chl (Figure 3C,D); for example, both PEG‐3 and Tween 20‐modified nanovesicles showed similar DNA release even though these two additives represent the fastest release (PEG‐3) and the slowest release (Tween 20), respectively, among all tested formulations. This result suggests that the role of the nanoparticles in enhancing photosensitizer interaction with bacterial cells is more important than their capacity to control photosensitizer release, at least at the 1 h timescale studied in this experiment. In this context, nanovesicles with slower or even plateaued release kinetics (e.g., cholesterol) may still be advantageous for bacterial inactivation in addition to providing improved stabilizing effects toward the photosensitizer. Consistent with this hypothesis, the Type II cholesterol‐modified nanovesicles that enabled the highest encapsulation efficiency (Figure 3B) but slow release kinetics (Figure 3D) induced the highest DNA release among all the formulations tested. It should be emphasized that nanovesicle binding to the cell membrane may also destabilize the nanovesicle bilayer to stimulate enhanced local Mg‐chl release beyond the plateau values observed in Figure 3D in the absence of light stimulation [79], making the discrimination of whether encapsulated or released Mg‐chl is the true bioactive component in the cell tests challenging. The apparent co‐localization of cholesterol‐modified nanovesicles shown in Figure 5 may also contribute by further enhancing local singlet oxygen concentrations at specific locations around the cell.

#### 3.4.3 Antibacterial Efficacy

When sprayed in the field, agricultural formulations dry on leaf surfaces and remain dry until rain or morning dew re‐wets the leaf surface (as reviewed in Ref. [4]). To better mimic processes naturally occurring in the field following foliar spray, 20 μL of a bacterial suspension with a 5% fetal bovine serum soil load was mixed with either free or nanovesicle‐encapsulated Mg‐chl and exposed to intense light for 1 h until the droplet was completely dry. The results of this test are shown in Figure 6B. Free Mg‐chl showed minimal antibacterial activity (<1‐log reduction in bacterial viability) while Mg‐chl‐loaded nanovesicles enabled at least a 3‐log reduction in bacterial concentrations regardless of the modifier added, consistent with the DNA release results collected in suspension (Figure 6A). The cholesterol‐modified nanovesicle again performed the best among the modified nanovesicles, a result also consistent with the DNA release results. However, all modified nanovesicles killed less effectively than the modifier‐free nanovesicles, a result most likely attributed to the lower effective CTAB content in the modified nanovesicles, which has advantages in terms of reducing phytotoxicity. No correlation between Mg‐chl release kinetics (Figure 3C,D) and antibacterial efficacy (Figure 6B), again in parallel to the DNA release results and consistent with potential membrane reorganization/disruption upon drying the nanovesicles on a leaf surface. Indeed, the antibacterial activity observed with these formulations under simulated field conditions is likely enhanced by the drying of the spray‐based formulation on the bacteria, promoting faster release while at the same time increasing the proximity between the nanovesicles/photosensitizers and pathogens and enabling more efficient ROS utilization upon photoactivation.

#### 3.4.4 In Planta Penetration

**FIGURE 7:**
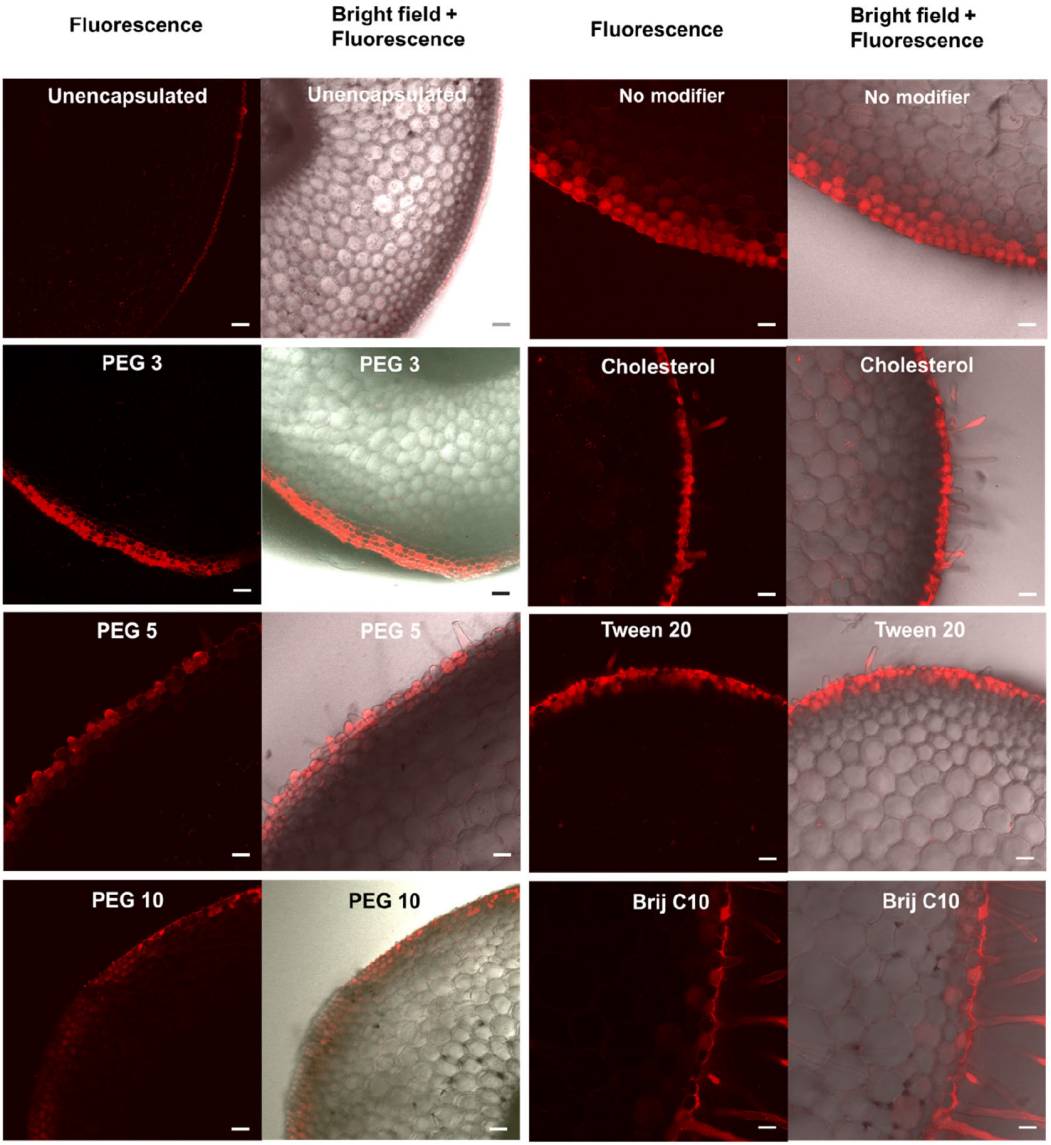
Penetration and cell uptake in soybean roots of free 0.1% (w/w) Mg‐chl and SDBS‐CTAB nanovesicles loaded with Mg‐chl (0.1% (w/w)) and prepared with or without modification with Type 1 (left column) and Type 2 (right column) modifiers (20 mol% modifier concentration) visualized in cross‐section by confocal microscopy. The red fluorescence indicates the presence of Mg‐chl (488 nm excitation wavelength). Scale bar = 20 μm in all images.

To assess the potential of the nanovesicles to enhance photosensitizer uptake into plants to more effectively treat plant disease, Figure 7 shows the penetration capacity of different nanovesicle formulations into soybean roots. Soybean roots were selected to avoid chlorophyll autofluorescence in leaves that overlaps with Mg‐chl fluorescence and thus complicates the analysis of nanoparticle uptake. Free Mg‐chl shows very limited penetration into the root, with the vast majority of the signal retained on the negatively charged root surface. In contrast, all nanovesicles enabled significantly improved retention and accumulation of the photosensitizer in the root relative to free Mg‐chl. Modifier‐free nanovesicles enabled effective penetration into 3–4 cell layers as well as efficient uptake into plant cells, consistent with the red coloration throughout the volume of cells within the penetration zone. Type I‐modified nanovesicles exhibited similar properties but even deeper penetration into the root, with PEG‐3 modified nanovesicles visible as deep as 8–10 cell layers into the root while PEG‐5 and PEG‐10 modified nanovesicles were visible at least 4 cell layers deep. In comparison, Type II‐modified nanovesicles exhibited lower penetration, with cholesterol and Brij C10‐modified nanovesicles penetrating only into the epidermis while Tween 20‐modified nanovesicles penetrated 2–3 cell layers deep. Despite this more limited penetration capacity, apparent cell uptake of Mg‐chl was still visible in the outer cell layer that could be accessed by nanovesicle transport, consistent with cholesterol promoting strong cell‐nanovesicle interactions as per the bacterial adhesion results in Figure 5. Of note, this difference in penetration has no correlation with the particle size of the nanovesicle; indeed, the PEG‐3 nanovesicle that showed the best overall penetration has the highest particle size among all nanovesicles tested (Figure 2A,B). Instead, the degree of nanoparticle penetration appears to be directly linked to the stiffness of the nanovesicle bilayer, with softer bilayers (Type I‐modified) able to penetrate more effectively while stiffer bilayers (Type II‐modified) slow down penetration [81].

Overall, both modifier‐free and Type I modified nanovesicles facilitate enhanced retention of the photosensitizer at the root, enhanced penetration into the root, and enhanced capacity for cell internalization of the photosensitizer relative to free Mg‐cln. In contrast, while Type II modified nanovesicles also significantly enhanced root retention and cell uptake, they offered more limited penetration benefits and mostly accumulated around the epidermis, although the cholesterol‐functionalized nanovesicles consistently showed the best antibacterial performance both in suspension and following drying on a surface. Given that both types of nanovesicles have been demonstrated to enable effective bacterial deactivation in both liquid suspension (Figure 6A) and in a dried droplet (Figure 6B), these result may be relevant to tuning the localization of high photosensitizer concentrations in different places within the plant, as may be necessary to treat different types of plant pathogens that may colonize either on or inside different parts of the plant [82].

## 4 Conclusions

Sodium dodecylbenzenesulfonate/cetyltrimethylammonium bromide (SDBS/CTAB) nanovesicles modified with additives that can either weaken or strengthen the nanovesicle membrane can effectively encapsulate and deliver the photosensitizer magnesium chlorophyllin (Mg‐chl). Mg‐chl can be loaded with high encapsulation efficiencies (>60%) into surfactant nanovesicles with tunable small particle sizes (∼90–170 nm). Modification with PEG phosphate ester surfactants containing unsaturated hydrophobes reduced the bilayer stability to enable faster and water‐triggerable Mg‐chl release (driven primarily by partitioning effects) tunable based on the PEG chain length, while modification with cholesterol and other saturated surfactants (e.g., Tween 20) improved the membrane stability and thus facilitated slower Mg‐chl release governed primarily by diffusion across the membrane. All nanovesicles enabled significantly improved association of the photosensitizer with bacterial membranes and, by extension, significantly higher light‐triggered bacterial killing reaching the level of effective disinfection (>3 log reduction in CFU count), with cholesterol‐modified nanovesicles in particular enhancing co‐localization of nanovesicles and bacteria as well as light‐activated DNA release in suspension and following drying on an interface mimicking practical foliar sprays in fields. Nanovesicles also significantly improved the retention of the photosensitizer in the root while also enabling enhanced cell uptake and tunable penetration into the root, depending on the bilayer stiffness, not the particle charge, enabling tunable degrees of bioactive penetration into plant roots depending on the target pathogen. Collectively, these results suggest the utility of nanovesicle‐based formulations as delivery vehicles for light‐activated anti‐pathogen agrochemicals, relevant for the delivery of photosensitizing agents and/or other functional bioactives for improved crop and plant protection.

## Author Contributions

**Lisha Zhao**: conceptualization (supporting), data curation (lead), formal analysis (lead), investigation (lead), methodology (lead), validation (lead), visualization (lead), writing – original draft (lead). **Wenzi Ckurshumova**: conceptualization (supporting), formal analysis (supporting), investigation (supporting), resources (supporting), writing – review and editing (supporting). **Ava Ettehadolhagh**: formal analysis (supporting), investigation (supporting), methodology (supporting), writing – review and editing (supporting). **Jun Liu**: conceptualization (equal), funding acquisition (equal), methodology (supporting), resources (supporting), supervision (supporting), writing – review and editing (supporting). **Michael Fefer**: conceptualization (equal), funding acquisition (equal), methodology (supporting), project administration (supporting), resources (supporting), supervision (supporting), writing – review and editing (supporting). **Todd Hoare**: conceptualization (equal), data curation (supporting), formal analysis (supporting), funding acquisition (equal), methodology (supporting), projectadministration (lead), resources (supporting), supervision (lead), writing – review and editing (lead).

## Supporting Information

Additional supporting information can be found online in the Supporting Information section.

## Funding

This work was supported by Mitacs (IT10216 and IT16650) and Suncor Energy Incorporated (Research Contract).

## Conflicts of Interest

The authors declare no conflicts of interest.

## Supporting information

Supplementary Material

## Data Availability

The data that support the findings of this study are available from the corresponding author upon reasonable request.
