# Supplementary material for: Nanovesicle‐Based Delivery of Magnesium Chlorophyllin for Photodynamic Inactivation in Agriculture
Source: Small Sci. 2026 May 8;6(5):e202500655. doi: 10.1002/smsc.202500655 (PMC13155147; doi:10.1002/smsc.202500655)
Supplement: Supplementary file 1 — Supplementary Material [file SMSC-6-e202500655-s001.pdf]

SUPPORTING INFORMATION

Nanovesicle-Based Delivery of Magnesium Chlorophyllin for Photodynamic Inactivation in Agriculture

Lisha Zhao[1], Wenzi Ckurshumova[2,3] , Ava Ettehadolhagh[1], Jun Liu[2], Michael Fefer[2,4], and Todd Hoare[1,*]

[1] Department of Chemical Engineering, 1280 Main Street West, Hamilton, Ontario, Canada L8S 4L7 – Lisha Zhao (zls04012023@gmail.com), Ava Ettehadolhagh (ettahada@mcmaster.ca), Todd Hoare (hoaretr@mcmaster.ca)

[2] Suncor Energy, 150-6 Avenue SW, P.O. Box 2844, Calgary, Alberta, Canada T2P 3E3 - Jun Liu (liujun77@yahoo.com)

[3] Centennial College, 941 Progress Avenue, Scarborough, Ontario, Canada M1G 3T8 - Wenzi Ckurshumova (Wckurshumova@centennialcollege.ca)

[4] Whitby Ag Consulting, Whitby, ON – Michael Fefer (MichaelFefer@WhitbyAgConsult.onmicrosoft.com)

* Corresponding author.

E-mail: hoaretr@mcmaster.ca (T. Hoare)

Tel.: (905) 525-9140 ext.24701

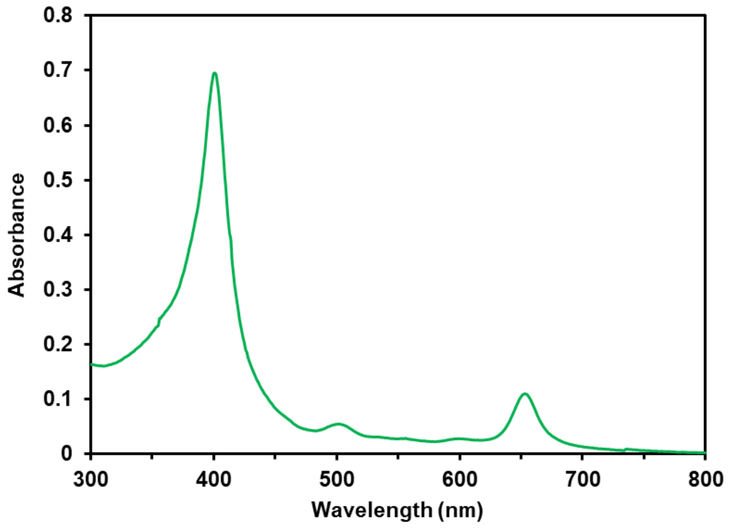

Figure S1. Full spectrum UV-vis scan of Mg-chl.

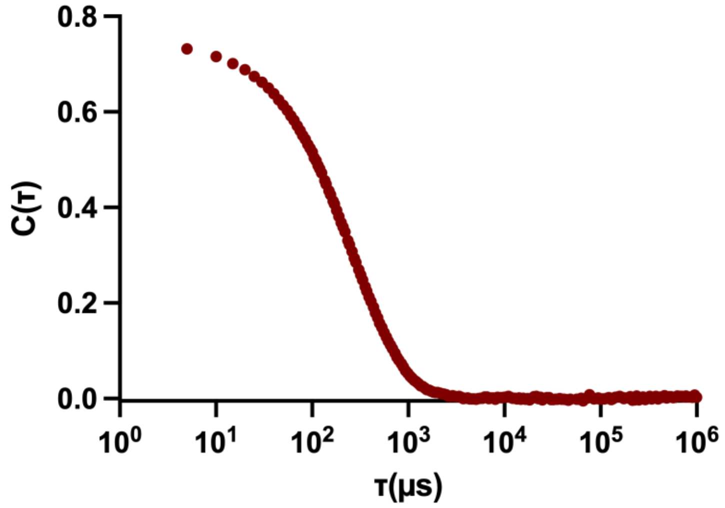

Figure S2. Dynamic Light Scattering (DLS) correlogram of Brij C10-modified nanovesicles. The autocorrelation function (G(τ)) is plotted as a function of time delay (τ, μs) on a logarithmic scale and shows the expected logarithmic decay for a good fit to the diffusion coefficient model.

Table S1. Comparison of particle sizes measured using dynamic light scattering (DLS) and transmission electron microscopy (TEM)

Material	DLS (nm)	TEM (nm)
No Modifier	92 ± 3	45 ± 7
PEG-3	179 ± 4	82 ± 16
PEG-5	132 ± 4	63 ± 7
PEG-10	96 ± 3	42 ± 9
Cholesterol	105 ± 4	79 ± 3
Tween 20	155 ± 5	111 ± 35
Brij C10	140 ± 4	86 ± 22
